# Draft genome sequence of *Lactiplantibacillus plantarum* KCKM 0106, isolated from mustard leaf kimchi

**DOI:** 10.1128/mra.00908-23

**Published:** 2023-12-01

**Authors:** Hye In Ko, So-Rim Kim, Chae-Rim Jeong, Moeun Lee, Jong-Bang Eun, Tae-Woon Kim

**Affiliations:** 1 Technology Innovation Research Division, World Institute of Kimchi, Gwangju, South Korea; 2 Department of Integrative Food, Bioscience and Biotechnology, Chonnam National University, Gwangju, South Korea; Department of Biology, Queens College, Queens, New York, USA

**Keywords:** whole-genome sequencing, probiotics, *Lactiplantibacillus plantarum*, kimchi, lactic acid bacteria, mustard leaf kimchi, KCKM, KACC

## Abstract

*Lactiplantibacillus plantarum* KCKM 0106, isolated from mustard leaf kimchi, demonstrates probiotic properties, such as acid tolerance and adhesion to intestinal epithelial cells. We present the draft genome sequence of *L. plantarum* KCKM 0106, comprising 3,328,662 bp and 44.4% GC content.

## ANNOUNCEMENT

*Lactiplantibacillus plantarum*, one of the main species of lactic acid bacteria and a probiotic candidate, is found in diverse environments, such as fermented foods and the gastrointestinal tract of animals and humans (1, 2). Mustard leaf components have been reported to exert antibiotic, antimicrobial, antioxidant, and antiatherogenic effects (3 – 6). *L. plantarum* KCKM 0106 (KACC 23090) was isolated from mustard leaf kimchi (Gat kimchi) at the World Institute of Kimchi (Gwangju, South Korea). The sample was serially diluted with 0.85% saline diluent (3M Company, St. Paul, MN, USA), and then plated on De Man Rogosa Sharp (MRS) agar, followed by incubation at 30°C for 48 h under anaerobic conditions. Finally, a single colony was streaked on MRS agar. The gene encoding the 16S rRNA of this bacteria was amplified using universal primers 27F (5ʹ-AGA GTT TGA TCM TGG CTC AG-3ʹ) and 1492R (5ʹ-TAC GGY TAC CTT GTT ACG ACT T-3ʹ), and the strain was identified as *L. plantarum* using 16S-based ID app from EzBioCloud.

To conduct whole-genome sequencing, *L. plantarum* KCKM 0106 was streaked on MRS agar and incubated at 30°C for 48 h under anaerobic conditions. Genomic DNA was extracted from colonies on the plate using Maxwell Prokaryote SEV DNA Purification Kit (Promega, Madison, WI, USA) according to the manufacturer’s instructions. Genomic DNA was sheared with Megaruptor 3 (Diagenode, Denvil, NJ, USA) and purified with AMPure PB (Pacific Biosciences, CA, USA). The library was sequenced using the Sequel II system (Pacific Biosciences, CA, USA) at Macrogen (Seoul, South Korea) and 75,395 HiFi reads (mean length, 8,611; *N*_50_, 9,115) were generated. The sequence was assembled using SMRTlink v11.0.0.146107 utilizing the Hierarchical Genome Assembly Process (7). For error correction, a library was prepared using the TruSeq Nano DNA High Throughput Library Prep Kit (Illumina, San Diego, CA, USA) in accordance with the manufacturer’s instructions. The NovaSeq platform (Illumina) was used for paired-end (2 × 150 bp) sequencing, which generated 13,336,950 raw and 9,536,684 filtered reads.

Illumina reads were trimmed using Trimmomatic 0.38 (8). Illumina reads were used to correct the assembly with Pilon v1.21 three times (9). BUSCO (v5.1.3) analysis was performed to assess assembly quality (10). Circular maps were generated using Circos v0.69.3 (11). *L. plantarum* KCKM 0106 consisted of one chromosome, two plasmids, and six contigs with a total length of 3,328,662 bp, *N*_50_ values of 3,110,977, GC content of 44.4%, and a depth of 194.9× (Table 1). The genome was annotated utilizing the Prokaryotic Genome Annotation Pipeline (PGAP 2023-10-03.build7061) (12). InterProScan v5.30-69.0 (13) and PSI-Blast v2.4.0 (14) with EggNOG DB v4.5 (15) were utilized for additional annotation. Finally, 3,152 coding sequences, 64 transfer RNAs, and 16 ribosomal RNAs were identified.

**TABLE 1:** Summary of the assembled draft genome sequence of *Lactiplantibacillus plantarum* KCKM 0106

	Contig 1	Contig 2	Contig 3	Contig 4	Contig 5	Contig 6	Contig 7	Contig 8	Contig 9	Total
Alias	Chromosome	Plasmid	Contig	Contig	Plasmid	Contig	Contig	Contig	Contig	
Length (bases)	3,110,977	55,886	52,851	40,595	23,475	14,571	10,521	9,955	9,831	3,328,662
GC (%)	44.7	39.0	42.7	39.7	42.7	38.6	36.7	38.3	36.6	44.4
Depth	192.4	313.8	263.7	239.0	214.6	72.9	115.8	74.0	71.9	194.9
CDS	2,813	61	43	40	22	14	15	9	11	3,028
tRNA	67	0	0	0	0	0	0	0	0	67
rRNA	16	0	0	0	0	0	0	0	0	16
Circular	Yes	Yes	Yes	Yes	Yes	Yes	Yes	Yes	Yes	–

## Data Availability

The associated draft genome sequences, BioProject, BioSample, and SRA accession numbers (PacBio and Illumina sequence reads) of *L. plantarum* KCKM 0106 are JAVKVE000000000, PRJNA1011495, SAMN37221010, SRR26065371, and SRR26446906.
